# Differences in cognitive shifts and prefrontal activation among athletes with different motor skills: an fNIRS study

**DOI:** 10.3389/fnhum.2026.1905190

**Published:** 2026-09-07

**Authors:** Tong Zhu, Yufei Liu, Su Wang, Chen Ning, Haolin Wang, Jiaxing Tian, Ying Qin, Liang Mu, Huayong Wu

**Affiliations:** 1 Harbin Institute of Physical Education, Harbin, Heilongjiang, China; 2 Jilin Institute of Physical Education, Harbin, Heilongjiang, China

**Keywords:** closed motor skills, cognitive switching, functional near-infrared spectroscopy, neural efficiency hypothesis, open motor skills, prefrontal cortex

## Abstract

**Objective:**

To investigate the effects of different motor skills (open and closed) on the cognitive switching function and hemodynamic response characteristics of the prefrontal cortex in male athletes, providing a neurocognitive basis for the scientific selection of high-level athletes and the development of personalized cross-training programs.

**Methods:**

This study recruited a total of 31 male Level 2 athletes, who were divided into an open-skill group (*n* = 16) and a closed-skill group (*n* = 15) based on the specific sports they had been practicing for a long time. Functional near-infrared spectroscopy (fNIRS) combined with a more-odd shift cognitive switching task was used to monitor changes in the oxyhemoglobin (HbO2) concentration in the dorsolateral prefrontal cortex (DLPFC), frontal pole (FPC), orbitofrontal cortex (OFC), and inferior frontal gyrus (IFG) in real time while the subjects performed the task in a natural state. Mixed-design ANOVA and independent samples T tests were used to systematically compare the behavioral performance (reaction time, accuracy, and switching cost) and brain region activation characteristics between the two groups.

**Results:**

1. Behavioral Results: Although there were no significant differences in overall accuracy or reaction time between the two groups, the interaction effect between group and condition was significant for reaction time. Under the premise of equal accuracy, the reaction time conversion cost of the open skill group (126.26 ± 92.26 ms) was significantly lower than that of the closed skill group (230.72 ± 143.03 ms), indicating higher cognitive conversion efficiency.2. HbO₂ Activation Results: During the cognitive conversion task, compared with the open skill group, the closed skill group had significantly higher HbO2 activation levels in the DLPFC (*p* = 0.001), FPC (*p* = 0.004), and OFC (*p* = 0.001). There was no significant difference in activation levels in the IFG between the two groups (*p* > 0.05). 3. HbR Activation Results: There were no significant intergroup differences in deoxygenated hemoglobin (HbR) levels across the four brain regions (IFG, DLPFC, FPC, and OFC) (*p* > 0.05)0.4. HbT Activation Results: Total hemoglobin (HbT) activation in the DLPFC (*p* = 0.008), FPC (*p* = 0.009), and OFC (*p* = 0.009) was significantly higher in the closed skill group than in the open skill group. There was no significant difference in HbT activation levels in the IFG between the two groups (*p* > 0.05)0.5. Correlation Results: Pearson correlation analysis revealed that there was no significant linear correlation between behavioral switching costs and cortical activation levels in any of the four brain regions for either the open or closed motor skill group (*p* > 0.05).

**Conclusion:**

Long-term open-skill training in dynamic, unpredictable contexts may enhance athletes’ cognitive flexibility and promote more efficient cortical resource allocation in critical cognitive control regions (DLPFC, FPC, and OFC). This study empirically validates the “neural efficiency hypothesis” and the “broad transfer hypothesis” from a neuroimaging standpoint. This study preliminarily indicates that moderate integration of open-skill cross-training into closed-skill programs may optimize the executive control network; however, the actual advantages of this method require validation through future extensive longitudinal intervention studies.

## 1 Introduction

Excellent athletic performance depends not only on athletes’ strong physical fitness and high technical levels but also on the significant role of their cognitive abilities (Alagoz et al., 2025). In recent years, researchers have increasingly explored the cognitive processes in sports (Walsh, 2014; Walton et al., 2018; Katwala, 2016). Studies have shown that athletes outperform the general population on certain cognitive tasks (Mann et al., 2007; Ong, 2015). Furthermore, an individual’s potential cognitive ability can predict future athletic achievements (Vestberg et al., 2017; Vestberg et al., 2012). Cognition refers to the abilities related to information acquisition, processing, utilization, and understanding, involving multiple functions such as attention, learning, memory, executive function, reasoning, and calculation (Harvey, 2019). Cognitive psychology divides cognitive abilities into basic and higher abilities (Liang et al., 2024). Basic cognitive abilities include attention, executive function, and memory, whereas higher cognitive abilities include textual reasoning, decision-making, and innovation (Gill et al., 2020; Miller and Cohen, 2001). Existing research has indicated that physical exercise can induce adaptive changes in brain structure and function and can influence the development of complex cognitive abilities (Yan et al., 2025; Gu et al., 2019). The degree of influence on cognitive abilities may be related to the characteristics of the sport being pursued.

On the basis of the influence of the task environment, motor skills can be divided into open motor skills and closed motor skills (Knapp, 2024). Open skills (such as basketball and soccer) occur in dynamic and unpredictable environments, requiring athletes to continuously respond to external cues, whereas closed skills (such as curling and shooting) occur in relatively static and predictable environments and are highly dependent on self-paced control, sustained internal focus, and strict inhibition of irrelevant distractions (Galligan, 2000). In complex and high-pressure sports environments, executive control, especially cognitive flexibility, is crucial for athletes to quickly adapt to rule changes and update their action strategies, and it is among the important factors affecting their athletic performance (Haugan et al., 2025). For example, athletes in open-skill sports must swiftly discard their initial strategies and adjust to new regulations during transitions between offense and defense, which necessitates significant cognitive flexibility; conversely, athletes in closed-skill sports demand prolonged concentration. The “broad transfer hypothesis” suggests that prolonged engagement in open-skill sports may improve overall cognitive flexibility; yet, study findings about the cognitive benefits of athletes across various skill kinds are still somewhat contentious. Certain behavioral studies have not demonstrated that open-skill athletes have a definitive advantage in all executive function subcomponents; some indicate that closed-skill athletes excel in particular response inhibition tasks or that there are no significant differences between the two groups. The discrepancies in research findings may stem from disparities in the complexity of cognitive tasks employed in various studies or from the inadequacy of relying exclusively on behavioral measures to uncover fundamental differences in neural processing. This necessitates a more thorough investigation of the underlying neurodynamic mechanisms. With the development of neuroimaging technology, functional near-infrared spectroscopy (fNIRS), with the advantages of being noninvasive, enabling real-time monitoring, and being resistant to motion interference, has become a core technical method for exploring the activation characteristics of the cerebral cortex. This technology has high ecological validity, enabling the real-time monitoring of prefrontal cortex hemodynamic changes in subjects performing tasks in a natural state. Nonetheless, the majority of prior fNIRS investigations concerning athletes’ executive skills have been confined to fundamental response inhibition tests (such as the Stroop or Go/No-Go tasks) and have not examined advanced cognitive switching and rule-reconfiguration capabilities. This study utilized the “more-odd shifting” challenge to fill this research gap. This exercise necessitates participants to often and swiftly alternate between various categorization rules, closely mirroring the cognitive challenges encountered by athletes in actual sports events, where they must abruptly modify their strategies in reaction to an opponent’s actions.

Cognitive switching flexibility is highly dependent on top-down regulation by the prefrontal cortex (Marotta et al., 2023). This study explicitly identified four crucial prefrontal regions of interest (ROIs) due to their functions being closely aligned with the higher-order cognitive requirements of competitiveness. Specifically, the dorsolateral prefrontal cortex is mainly responsible for working memory updating and rule reconstruction (Belluardo et al., 2025),and the frontal cortex participates in the coordination of multiple task goals,18 while the orbitofrontal cortex and inferior frontal gyrus play key roles in response inhibition and conflict resolution (Shao and Lee, 2017). In exploring the neural mechanisms of expert brains, the field of sports psychology has proposed the “neural efficiency hypothesis.” This hypothesis suggests that individuals with high skill levels or superior cognitive control can achieve better or equivalent behavioral performance with less cortical resource consumption (i.e., lower brain activation levels) when they complete tasks of the same difficulty (Buchsbaum et al., 1988). Therefore, in this study, fNIRS technology combined with a more-odd shift task was used to systematically compare the behavioral performance and activation characteristics of the prefrontal cortex (DLPFC, FPC, OFC, and IFG) of male athletes with open and closed skills when they performed rule transitions. This study aims to reveal the neurocognitive characteristics of athletes with different skills, provide new indicators for the selection of high-level athletes, construct a multidimensional selection system covering brain neural characteristics, and provide a precise basis for the formulation of personalized intervention programs. On the basis of the above theoretical review, this study proposes the following hypotheses: (1) At the behavioral level, open-skill athletes have better rule reconstruction and cognitive transition abilities because of long-term adaptation to the dynamically changing sports environment and therefore have lower transition costs and higher transition efficiency in transition tasks. (2) At the neurological level, when performing the same cognitive transition task, compared with closed-skill athletes, open-skill athletes will show significantly lower activation levels in key prefrontal cortex regions, resulting in more economical and efficient cortical neural network processing characteristics.

## 2 Materials and methods

### 2.1 Research subjects

Prior power analysis was performed using G*Power 3.1 software. With a significance level of α = 0.05 and a moderately large effect size (*f* = 0.27), a total sample size of at least 30 participants was required to achieve 80% statistical power. This study ultimately included 31 participants, meeting the statistical power requirements. In this study, 31 male Level 2 athletes were selected from Harbin Sport University. Participants were categorized into an open-motor-skill group (*n* = 16) and a closed-motor-skill group (*n* = 15) according to the specific sport they had been practicing for an extended duration. In this study, “Second-Level Athletes” denotes persons designated as “National Second-Level Athlete” by the General Administration of Sport of China. This classification necessitates that athletes meet established performance standards or rankings in certain events at provincial or national official sports contests. This inclusion criterion guarantees that the sample chosen for this study exhibits a high, consistent, and officially recognized level of professional athletic proficiency. There were no significant differences between the two groups in terms of age, height, weight, or years of training (*p* > 0.05), as detailed in Table 1. All participants met the following inclusion criteria: (1) normal visual acuity or corrected visual acuity and normal color vision; (2) confirmation of being right-handed via the Edinburgh Hand Disposition Scale; and (3) no history of neurological or psychiatric illness and no recent use of any psychotropic drugs. Before the experiment began, all participants were fully informed of the experimental procedure and signed informed consent forms. The design and implementation of this study were approved by the Ethics Committee of Harbin Sport University (2025334) and strictly followed the guiding principles of the Declaration of Helsinki.

**Table 1:** Basic information on participants.

Group	*n*	Age(years)	Height(cm)	Weight(kg)	Training years
Open-skill	16	20.20 ± 1.014	179.8 ± 3.448	80.1 ± 2.999	7.20 ± 1.082
Closed-skill	15	20.75 ± 1.291	180.3 ± 3.114	80.4 ± 2.502	7.38 ± 1.147
*P*	—	0.152	0.667	0.975	0.666

### 2.2 Experimental apparatus

**Figure 1:**
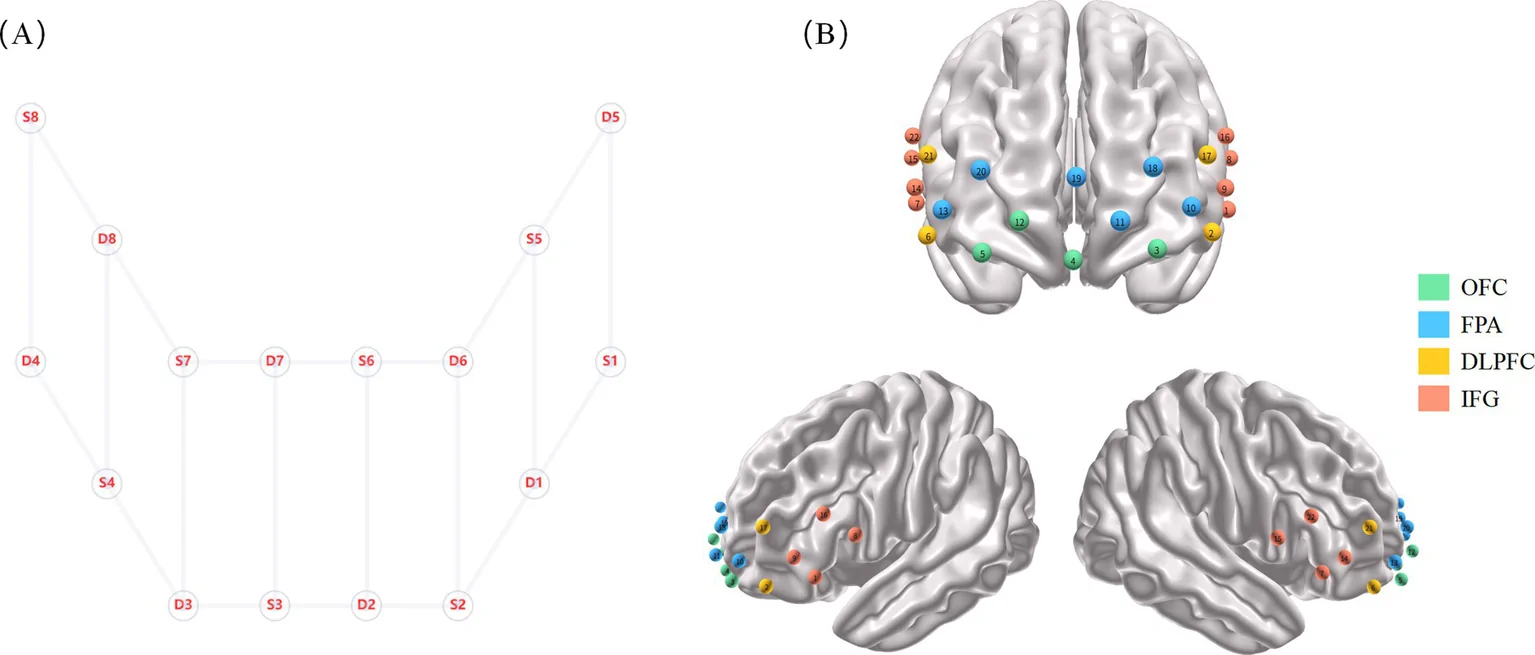
**(A)** Two-dimensional layout of the photoelectrodes. **(B)** The 22 channels are distributed across four regions of interest (ROIs), corresponding to four anatomically symmetrical brain regions: the orbitofrontal cortex (OFC), the frontal pole area (FPA), the dorsolateral prefrontal cortex (DLPFC), and the inferior frontal gyrus (IFG).

In this study, a NirLight-300A device (Wuhan Yiruide Group, Wuhan, China) was used for data acquisition. The system employs dual-wavelength laser diodes (690 nm and 830 nm), with a sampling frequency of 20 Hz, and is configured with 8 emitters and 8 detectors, forming 22 channels. The polarity arrangement is shown in Figure 1. Following the internationally accepted 10–20 system, emitter S2 corresponds to the Fpz point. The spatial coordinates of the reference points (Nz, Cz, AL, and RL) and all the polarities were recorded using a 3D localizer. Spatial localization was performed using the NIRS-SPM method13, accurately projecting the channels onto the cortical surface and matching them to the corresponding Brodmann regions (see Figure 1). ROIs, including the OFC, FPC, DLPFC, and IFG, were selected on the basis of the Brodmann partitioning system. The corresponding channels for each ROI are shown in Table 2.

**Table 2:** Channel information for each brain region.

Channel ID	X	Y	Z	BA	Coverage (%)	Channel ID	X	Y	Z	BA	Coverage (%)
Ch01	-55	35	-3	IFG	65.9	Ch12	17	73	-2	OFC	50.8
Ch02	−45	54	−8	DLPFC	68	Ch13	42	62	0	PA	66.4
Ch03	−25	67	−11	OFC	95.3	Ch14	56	40	5	IFG	83.7
Ch04	1	68	−14	OFC	83.1	Ch15	64	13	14	IFG	33.9
Ch05	29	67	−12	OFC	85.8	Ch16	−55	31	22	IFG	97.9
Ch06	49	53	−9	DLPFC	68.2	Ch17	−43	54	16	DLPFC	84.4
Ch07	58	31	−1	IFG	69.4	Ch18	−23	70	13	FPA	99.3
Ch08	−60	19	14	IFG	41.5	Ch19	0	68	10	FPA	100
Ch09	−52	43	5	IFG	65.1	Ch20	29	68	12	FPA	94.7
Ch010	−36	64	1	FPA	77.4	Ch21	49	50	16	DLPFC	66.5
Ch11	−13	73	−2	FPA	57.1	Ch22	60	26	22	IFG	69

IFG, Inferior frontal gyrus; DLPFC, Dorsolateral prefrontal cortex; OFC, Orbitofrontal area.

FPA, Frontopolar area.

### 2.3 Experimental procedure

The more-odd shift task was used to assess participants’ cognitive flexibility. The experimental procedure was written and presented using E-Prime 3.0 software (Psychology Software Tools, Pittsburgh, PA, United States). During the task, a series of single-digit numbers (1, 2, 3, 4, 6, 7, 8, and 9) were displayed in the center of the computer screen. The task rules depended strictly on the color of the presented numbers: Size judgment task (red numbers): Participants were asked to judge whether the number was greater than or less than 5. If it was greater than 5, they pressed the “F” key; if it was less than 5, they pressed the “J” key. Parity judgment task (green numbers): Participants were asked to judge whether the number was odd or even. If it was odd, they pressed the “F” key; if it was even, they pressed the “J” key.

**Figure 2:**
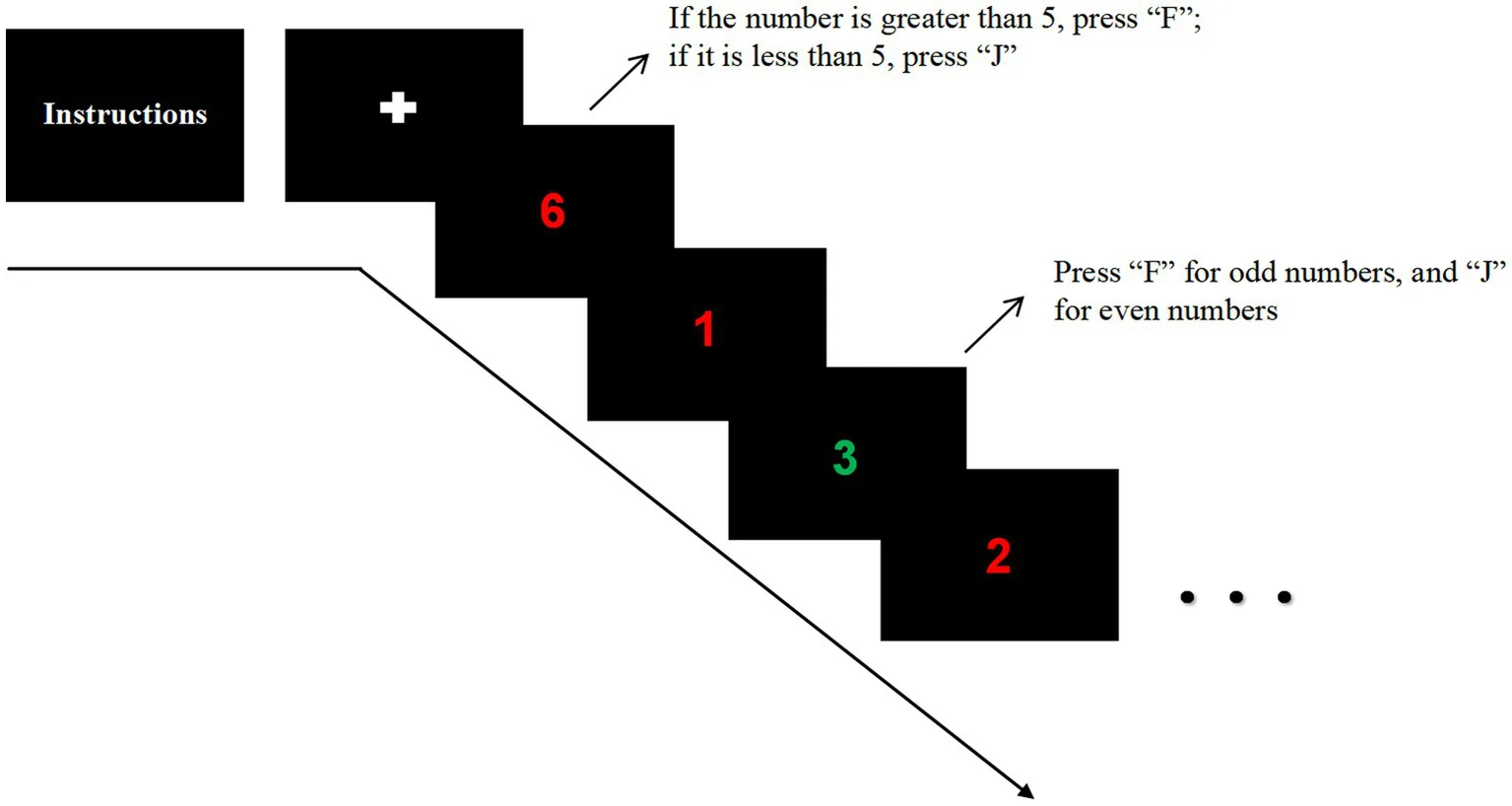
More-odd shifting task flow.

The experiment commences with a practice module initiated by hitting the “Enter” key to ensure participants comprehensively grasp the task instructions and key mappings. Participants must attain a minimum accuracy rate of 80% during the practice phase to advance to the main trial. After the practice, the formal experiment commenced, consisting of three blocks, each containing 36 trials, for a total of 108 trials. Each trial commences with a “+” symbol positioned centrally on the screen, followed by the display of the target stimulus, which persists until the participant answers or the maximum presentation duration of 2000 ms elapses, after which it vanishes. A constant inter-trial interval ensues after the cessation of each stimulus. Trials were categorized into two conditions: “repeat trial” (the current trial rule is the same as the previous trial) and “switch trial” (the current trial rule is different from the previous trial). This experiment implemented several control techniques to rigorously mitigate order effects and tiredness effects. A brief 5-min intermission was allocated between each of the three test blocks; additionally, the probabilities for the size judgment and odd-even judgment tasks were established as equal, and within each block, the sequence of repeated and switched trials was administered in a pseudorandom fashion to avert strategic anticipation effects among participants. The more-odd shift task flow is shown in Figure 2.

### 2.4 fNIRS data processing

This research employed the Nirsmart toolset for the preprocessing and analysis of fNIRS data. The initial raw light intensity signals were transformed into optical density. To mitigate motion artifacts, time frames exhibiting abrupt, high-amplitude fluctuations were automatically detected, followed by the application of strip interpolation to rectify the impacted segments, thus diminishing the influence of transient disturbances. Thereafter, alterations in the concentrations of oxygenated hemoglobin, deoxygenated hemoglobin, and total hemoglobin were determined using the modified Beer–Lambert law.

This study employed block averaging and feature extraction to achieve a steadier concentration response. Analysis segments were extracted from 9 s prior to the task to 58 s subsequent to the task, based on each task marker. For each segmented data set, the mean value of the baseline period (−9 s to −0.1 s) preceding the relevant task marker was initially subtracted to achieve baseline correction. To enhance the signal-to-noise ratio and meticulously manage artifacts, the system executed a coefficient of variation (CV)-based outlier removal procedure at this juncture, systematically excluding invalid trials characterized by poor signal quality and excessive variability, even post-interpolation correction. Thereafter, block-averaged concentrations were derived by averaging the corrected segmented data.

This study utilized a generalized linear model (GLM) to derive statistical measures of task-related activity in brain regions. The model regarded the observed hemodynamic signals as the dependent variable and represented them as a linear combination of experimental circumstances according to the traditional hemodynamic response function (HRF). The *β* values derived from parameter estimation indicated the magnitude of task-induced activation and were extracted for further population-level statistical analysis. To mitigate experimenter bias, this study employed analyst blinding during the data processing and analysis stages. Researchers engaged in data preprocessing, artifact elimination, and statistical analysis remained unaware of participant group designations during the whole procedure, and all data were processed independently utilizing anonymized identities.

### 2.5 Statistical analysis

#### 2.5.1 Behavioral statistical analysis

The behavioral metrics extracted from E-Prime included total accuracy (ACC), reaction time on the repeat (RT Repeat), and reaction time on the switch (RT Switch). In the reaction time analysis, trials with excessively fast reactions (RT < 200 ms), timeouts, and incorrect key presses were excluded. The core behavioral metric for cognitive flexibility was Switch Cost, which is the difference between the reaction time of a switch trial and a repeat trial (RT Switch—RT Repeat). A mixed-design ANOVA with 2 (groups: open vs. closed skill groups) × 2 (conditions: repeat vs. switch) was conducted using GraphPad Prism 10.0 software.

### 2.6 fNIRS data analysis

Statistical analysis was performed using GraphPad Prism 10.0 software. The normality of the brain activation data was verified by the Shapiro–Wilk test, and the homogeneity of variance was confirmed by the Levene test. The data are expressed as the mean ± SD. Independent samples T tests were used for intergroup comparisons. For data that did not conform to a normal distribution, the Mann–Whitney U test was used for intergroup comparisons. Multiple hypothesis testing between regions of interest (ROIs) was adjusted using the false discovery rate (FDR) method. All statistical results in this experiment were adjusted for FDR. To further assess the practical significance of differences between groups, effect sizes were expressed as Hedges’ g and 95% bootstrap confidence intervals.

## 3 Results

### 3.1 Analysis of Behavioral results

**Figure 3:**
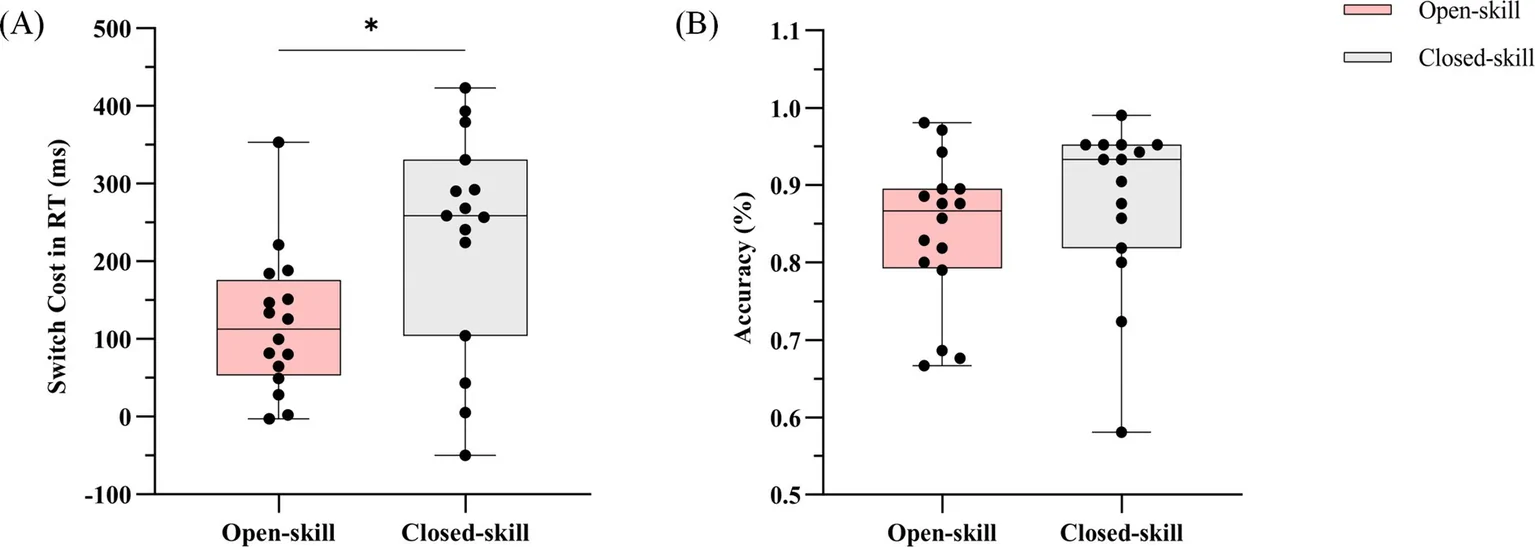
Behavioral results of the two groups of athletes completing the more-odd shifting task: **(A)** Comparison of switching costs between the two groups. **(B)** Comparison of accuracy rates between the two groups. *p < 0.05; All *p*-values have been FDR-corrected.

The results revealed that the main effects of the conditions were extremely significant for both reaction time and accuracy. Compared with the repeated conditions, the overall reaction time under the changed conditions was significantly longer [*F*(1, 29) = 67.863, *p* < 0.001, $\eta_{p}^{2}$ = 0.701], and the overall accuracy was significantly lower [*F*(1, 29) = 39.321, *p* < 0.001, $\eta_{p}^{2}$ = 0.576]. The main effects of group were not significant for either reaction time [*F*(1, 29) = 0.013, *p* = 0.910, $\eta_{p}^{2}$ < 0.001] or accuracy [*F*(1, 29) = 0.433, *p* = 0.516, $\eta_{p}^{2}$ = 0.015]. However, the interaction effect between group and condition was not significant for accuracy [*F*(1, 29) = 0.408, *p* = 0.528, $\eta_{p}^{2}$ = 0.014]. The interaction was significant in terms of reaction time [*F*(1, 29) = 5.917, *p* = 0.021, $\eta_{p}^{2}$ = 0.169]. Further analysis of switching costs revealed that the switching cost of the open skill group (126.26 ± 92.26 ms) was significantly lower than that of the closed skill group (230.72 ± 143.03 ms). The behavioral results indicate that while maintaining the same accuracy, athletes with open skills exhibited relatively lower switching costs and higher switching efficiency in cognitive switching tasks (see Figure 3).

### 3.2 Analysis of brain region activation results

#### 3.2.1 Analysis of HbO₂ activation results in brain regions

**Figure 4:**
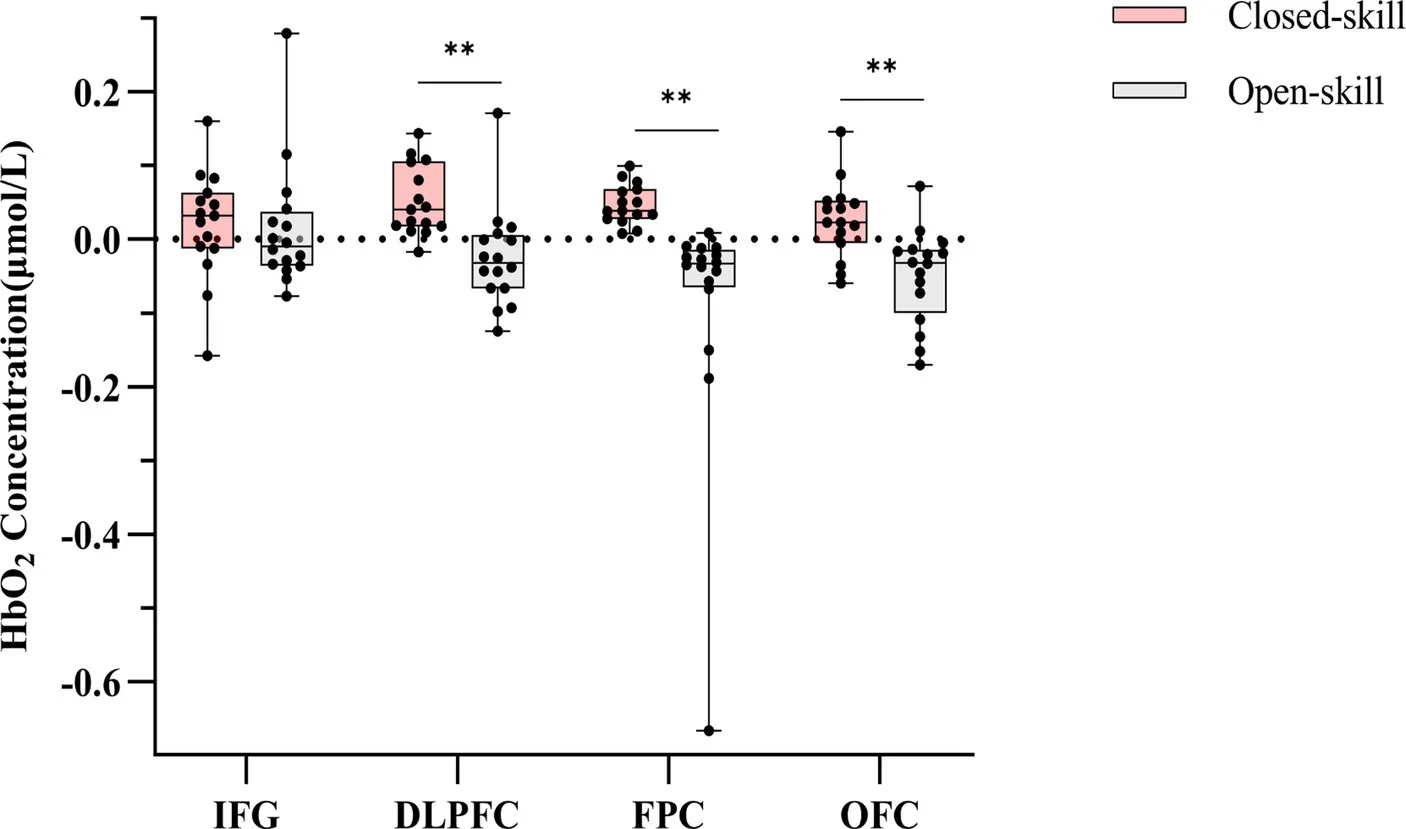
Comparison of *ẞ* values for oxygenated hemoglobin concentration during the more-odd shifting task; *P < 0.01; all *P*-values have been FDR-corrected.

A comparison examination of HbO₂ activation levels in designated brain areas among athletes in the open-set and closed-set groups during the “more-odd” shifting task indicates, as illustrated in Figure 4, that the HbO2 activation levels in the dorsolateral prefrontal cortex [*p* = 0.001, 95% CI (0.034, 0.121), *Hedges’ g* = 1.278], frontal pole [*p* = 0.004, 95% CI (0.046, 0.221), *Hedges’ g* = 1.091], and orbitofrontal cortex [*p* = 0.001, 95% CI (0.033, 0.120), *Hedges’ g* = 1.266] were significantly greater in the closed group than in the open group. There was no significant difference in activation levels between the two groups in the inferior frontal gyrus (*p* > 0.05).

#### 3.2.2 Analysis of HbR activation results in brain regions

**Figure 5:**
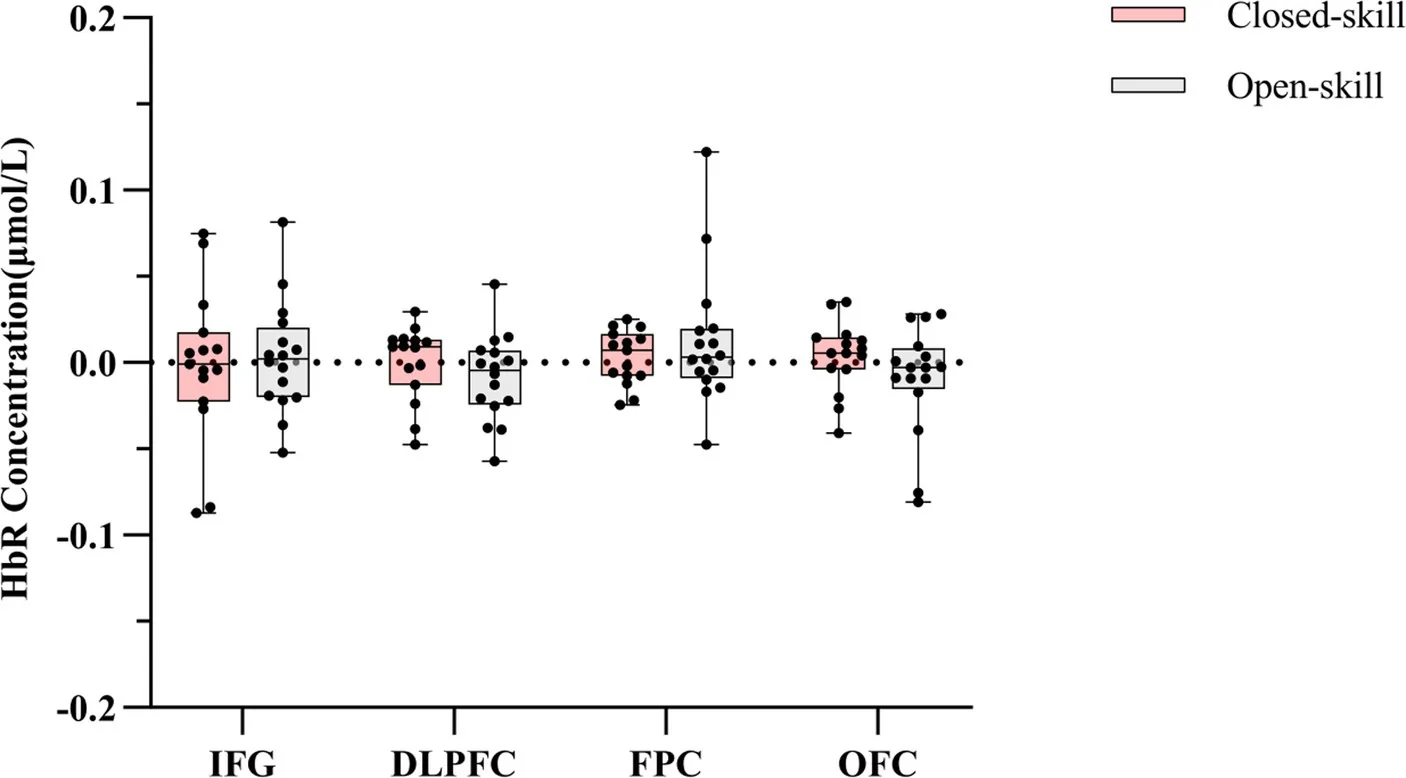
Comparison of *ẞ* values for deoxyhemoglobin concentration during the more-odd shifting task.

A comparative analysis of HbT activation levels in brain regions during the “more-odd shifting” task between the open-group and closed-group participants showed that, as illustrated in Figure 5, there were no significant differences between the two groups in the inferior frontal gyrus, dorsolateral prefrontal cortex, frontal pole, and orbitofrontal cortex (*p* > 0.05).

#### 3.2.3 Analysis of HbT activation results in brain regions

**Figure 6:**
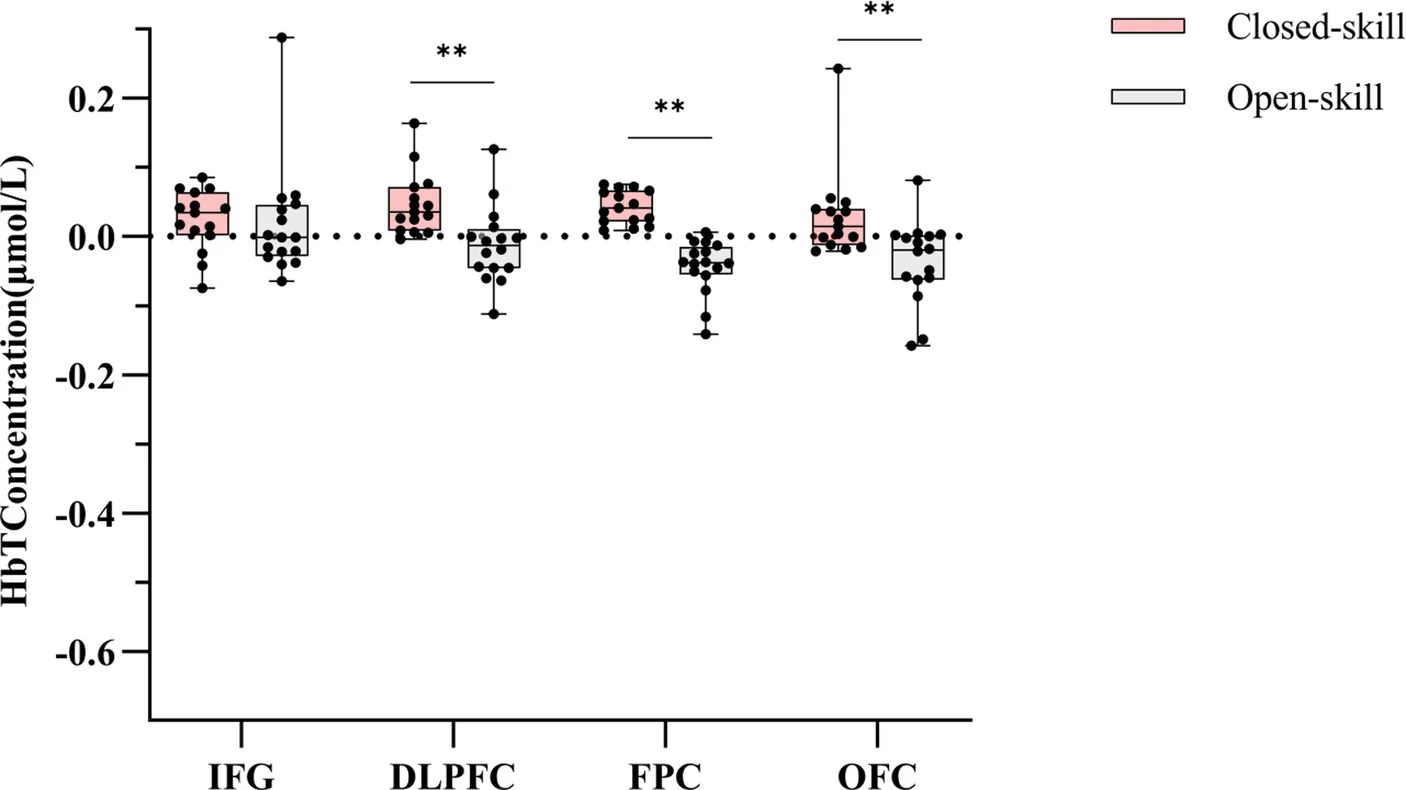
Comparison of *ẞ* values for oxygenated hemoglobin concentration during the more-odd shifting task; *P < 0.01; all *P*-values have been FDR-corrected.

A comparative analysis of HbO₂ activation levels in specific brain regions during the “more-odd shifting” task between the open-book and closed-book groups shows, as illustrated in Figure 6, that the closed-book group exhibited significantly higher activation in the dorsolateral prefrontal cortex [*p* = 0.008, 95% CI (0.022, 0.096), *Hedges’ g* = 1.139], the frontal pole [*p* = 0.009, 95% CI (0.063, 0.111), *Hedges’ g* = 2.568], and the orbitofrontal cortex [*p* = 0.009, 95% CI (0.020, 0.111), *Hedges’ g* = 1.021] were significantly higher than those in the open-access group. In the inferior frontal gyrus, there was no significant difference in activation levels between the two groups (*p* > 0.05).

### 3.3 Correlation analysis between behavioral performance and brain activation

To further explore the relationship between cognitive switching efficiency and prefrontal hemodynamics, we conducted a Pearson correlation analysis of behavioral switching costs and oxygenated hemoglobin levels in four brain regions for the open motor skill group and the closed motor skill group. The results showed that there was no significant linear correlation between switching costs and cortical activation levels in any of the four brain regions for either group (*p* > 0.05) (see Supplementary Figure S1 for detailed scatter plots).

## 4 Discussion

In this study, functional near-infrared spectroscopy (fNIRS) combined with a more-odd shift task paradigm was used to systematically examine the behavioral performance and prefrontal cortex hemodynamic response characteristics of male athletes with open and closed skills during advanced rule transitions. After the speed-accuracy tradeoff was excluded, open-skill athletes exhibited significantly lower reaction time transition costs. Hemodynamically, compared with closed-skill athletes, open-skill athletes showed significantly lower activation levels in the dorsolateral prefrontal cortex (DLPFC), frontal pole (FPC), and orbitofrontal cortex (OFC), indicating a low-activation-high-performance pattern.

In terms of behavioral aspects, the results of this study indicate that both groups of subjects had significantly slower reaction times under the switching condition than under the repetition condition, and their accuracy rates also decreased significantly, resulting in switching costs (Capizzi et al., 2020). However, the interaction between groups revealed that the switching costs of the open-skill group were significantly lower than those of the closed-skill group, demonstrating more efficient cognitive switching. These results are consistent with previous findings, namely, that athletes who have long been engaged in open-skill sports tend to outperform closed-skill athletes in cognitive tasks involving rapid reaction, rule switching, and inhibitory control. A review of previous studies by Koch et al. revealed that open-skill athletes had better working memory and cognitive flexibility than closed-skill athletes did, and athletes who trained in open-skill sports before the age of 18 performed better in both areas (Koch and Krenn, 2021). Furthermore, a review by Lai et al. revealed that open skills have a positive effect on inhibitory control and cognitive flexibility in children, adolescents, and adults, primarily manifested as shorter reaction times in inhibitory control tasks and shorter reaction times and lower switching costs in cognitive flexibility tasks (Lai et al., 2024). According the broad transfer hypothesis, long-term practice of specific skills can improve individuals’ cognitive abilities in situations outside of specific sports (Formenti et al., 2019). Despite an increasing interest in vision training for sport performance, whether it may be transferred to sport-specific skills and whether such transfer could be mediated by cognition remain open issues. To clarify this point, we tested the effects of 6-week sport vision training programs (requiring generic or volleyball-specific motor actions) in a non-sport-specific context and compared them to those of a third group of students who performed traditional volleyball training in a sport-specific context. Fifty-one female volleyball players were randomly assigned to one of three groups. Before and after the training period, the accuracy of the subjects’ volleyball-specific skills and cognitive performance (clinical reaction time, executive control, and perceptual speed) were tested. The accuracy of volleyball-specific skills improved after traditional volleyball training with respect to the vision training groups. Conversely, compared with the traditional volleyball training group, the vision training group had improved cognitive performance (clinical reaction time, executive control and perceptual speed). Our results have shown that vision training in a non-sport-specific context (either generic or with specific motor actions) improved cognitive performance but seemed to be less effective for improving sport-specific skills. This evidence suggests that the environment in which exercise is performed plays a key role in improving perceptions and actions related to sport-specific skills, supporting the ecological approach to sport learning. Multiple studies have shown that when athletes regularly participate in certain types of sports over a long period, the various cognitive demands of these sports can have different effects on executive function (Ballester et al., 2019; Becker et al., 2018; Chang et al., 2017; Jacobson and Matthaeus, 2014). Open-skill sports environments are characterized by high unpredictability, time pressure, and rapidly changing situations, requiring athletes to continuously engage in perceptual-motor coupling, make rapid decisions, and flexibly switch between multiple potential action options.30 This long-term, high-intensity environmental adaptation training may promote the neuroplasticity of the executive control network of the brain and optimize the efficiency of task set transitions. In contrast, closed-skill sports environments are relatively stable and predictable, with highly procedural and repetitive movement patterns. While extremely high precision and consistency in movement execution are needed, the need for rapid cognitive transitions is relatively low (Allard and Burnett, 1985). Therefore, the cognitive advantage of closed-skill athletes may be reflected more in sustained attention and movement sequence learning than in rapid cognitive flexibility. Thus, although the movement execution of closed-skill athletes is highly automated, their transition efficiency may be worse than that of open-skill athletes when they face cognitive challenges requiring rapid rule transitions.

Compared with athletes in the open skill group, athletes in the closed skill group showed stronger activation in regions related to greater cognitive control, such as the DLPFC, FPC, and OFC. The differences in activation levels in different brain regions between the two groups can be explained from different perspectives. First, in the cerebral cortex, the DLPFC and FPC are core hubs responsible for working memory updates and multitasking coordination, whereas the OFC plays a crucial role in error monitoring and conflict inhibition (Jones and Graff-Radford, 2021; Buss and Spencer, 2018; Asanowicz et al., 2021). The “low activation–high performance” pattern identified in open-ended skill sets strongly corroborates the “neural efficiency hypothesis. This hypothesis asserts that, through experience-dependent neuroplasticity, individuals engaged in prolonged training within cognitively demanding environments will optimize their pertinent neural circuits, allowing for more efficient neural activity during equivalent tasks with reduced neural resource utilization (Buchsbaum et al., 1988). Current study findings also corroborate this perspective. The results of the research by Guo et al. (2017) indicate that table tennis players show lower neural activation in task-related brain regions during go/no-go visuospatial tasks (Buchsbaum et al., 1988). This increased neural efficiency manifests as a bidirectional attenuation phenomenon, i.e., reduced activation in areas related to task execution but simultaneously reduced deactivation in areas related to the processing of irrelevant information (Qiu et al., 2019). fNIRS is an optical measurement based on the hemodynamic response of oxyhemoglobin and deoxyhemoglobin to neural activity (Lloyd-Fox et al., 2010) and is capable of measuring signals related to neural activity (Allison et al., 2013). This study reveals that the sustained high activation in the closed-skill group across three brain regions suggests that, when confronted with unfamiliar dynamic rule changes, the group—devoid of efficient cortical network support—was compelled to deploy substantial top-down cognitive control resources (evidenced by a significant increase in HbO₂ concentration in the prefrontal cortex) to counter impulsivity and sustain accuracy. In contrast, owing to the neuroadaptation shaped by a long-term open environment, the open skill group was able to efficiently complete rule transitions with lower activation levels. The results of this investigation concerning deoxyhemoglobin and total hemoglobin in the two groups further illustrate this trend of brain efficiency. Despite the absence of a significant difference in HbR between the two groups, athletes in the closed-circuit group exhibited markedly elevated total hemoglobin concentrations in the DLPFC, FPC, and OFC brain areas relative to those in the open-circuit group. Given that HbT serves as a dependable physiological marker of local total cerebral blood volume, the notable simultaneous rise in both HbO₂ and HbT reduces the impact of systemic physiological noise, thereby affirming at a multi-indicator level that closed-skill athletes enhance blood flow and metabolic resources in local cortical areas when adjusting to swift rule alterations. Secondly, although certain research has indicated that various sports may exert distinct influences on neurocognitive function, our study did not explicitly see alterations at the molecular level. Previous studies suggest that this may be associated with variations in the secretion of specific biomarkers within the neurochemical system. (Neepser et al., 1995; Liu-Ambrose et al., 2010; Cassilhas et al., 2012). Brain-derived neurotrophic factor (BDNF) and cathepsin B (CTSB) are key peripheral factors that regulate the relationship between physical exercise and cognition. Studies have shown that a rich environment can promote the expression of brain-derived neurotrophic factor (BDNF) (Cao et al., 2010), and compared with closed skill training, acute open skill training increases BDNF secretion (Hung et al., 2018). Evrim et al. found that, regardless of whether it was long-term exercise or a single exercise session, cathepsin B (CTSB) was significantly increased only in the open skill group (Gökçe et al., 2021) Exercise promotes brain plasticity by activating signaling pathways at the cellular and molecular levels (Aguiar et al., 2011). Central myelination is an important component of plasticity, and different types of exercise stimulate different changes in motor neural circuits (Garcia et al., 2012). Cognitively demanding exercises can promote the survival of neurons in the hippocampus, enhance connectivity in key white matter pathways, and promote myelination in related cortical regions (Raichlen and Alexander, 2017). Because open-skill exercises demand higher cognitive abilities, they may increase central myelination, which may explain why athletes in the open-skill group have lower activation levels in these three brain regions. Despite notable disparities among groups, this investigation found no significant linear association between individual behavioral switching costs and local HbO₂ activation in any group. The absence of a direct association at the individual level may be ascribed to the significant homogeneity of the sample in this study. Moreover, the phenomenon of “neural efficiency” at the individual level may not merely present as a linear reduction in absolute local blood oxygen content, but instead as enhanced functional connectivity or dynamic network coupling among various brain regions. Consequently, subsequent research should integrate methodologies like whole-brain functional connectivity analysis to elucidate the network-level neural mechanisms that underpin athletes’ cognitive benefits.

This study integrated behavioral testing with fNIRS technology to examine the potential correlation between varying levels of motor skill expertise and cognitive switching function, alongside hemodynamic properties of the prefrontal cortex, in male athletes. Prolonged engagement in open-skill training within dynamic and unpredictable environments may correlate with enhanced cognitive flexibility in athletes and facilitate the formation of more efficient cortical resource allocation patterns in critical cognitive control areas, including the DLPFC, FPC, and OFC. This “high performance–low consumption” neurocognitive characteristic provides empirical support with high ecological validity for the “neural efficiency hypothesis” and the “widespread transfer hypothesis” from a neuroimaging perspective, further demonstrating that complex sports environments can optimize the processing efficiency of the brain’s executive control network by inducing more efficient changes in neuroplasticity. This study provides not only a new perspective for exploring the neural adaptation mechanisms of expert-level brains but also potential objective neurobiological indicators for the scientific selection of future high-level athletes. The outcomes of this investigation possess exploratory significance in practical application. The neural efficiency traits in the prefrontal cortex may work as possible markers for evaluating athletes’ cognitive capacity in the future; nevertheless, this necessitates further validation through comprehensive longitudinal research. The findings indicate that, due to the relatively stable and predictable nature of closed-skill sports environments, the moderate integration of cross-training—which entails high cognitive demands and open-ended attributes—into daily training regimens may mitigate the constraints of closed-skill training alone in enhancing rapid cognitive switching. This may assist athletes in closed-skill sports in enhancing their capacity to withstand distractions and adjust flexibly in intricate competitive scenarios, such as erroneous referee decisions, unexpected noise, or tactical alterations. Nonetheless, it is crucial to underscore that it remains premature to convert these findings, derived from cross-sectional studies, into applicable talent selection criteria or training protocols. Future research must promptly implement large-sample, large-scale longitudinal intervention designs to rigorously assess the validity of these neurobiological indicators and the genuine causal effects of cross-training interventions.

## 5 Limitation

Although this study provides evidence at the neurological level regarding the influence of motor skill types on cognitive switching, extreme caution must be exercised when drawing causal inferences from these findings and generalizing them broadly. First, this study employed a cross-sectional design, which cannot establish a definitive causal relationship between motor skill experience and neurocognitive remodeling. Future research should conduct long-term longitudinal controlled trials to further validate the dynamic shaping process of the brain’s executive control network by different motor interventions. Secondly, the sample comprised solely 31 male elite athletes, representing a rather homogeneous cohort, and did not include a control group of non-athletes. This not only restricts the generalizability of the study’s findings to other populations (such as female athletes or the general public) but also hinders our capacity to ascertain whether athletes’ absolute cognitive performance is considerably superior to that of the general population. This study could not completely address nuanced variations in potential confounding variables, like absolute training volume and training intensity, among various sports. Furthermore, due to the limited penetration depth of fNIRS technology, this study examined only hemodynamic changes in the superficial prefrontal cortex and was unable to reveal the synergistic roles of deeper structures, such as the basal ganglia or cerebellum, in rule switching. Finally, although this study employed classic computerized cognitive tasks with good internal validity, its ecological validity may be insufficient to fully simulate the complex and high-pressure dynamic environment of real-world competitive sports. Future research could explore the integration of virtual reality (VR) technology or the use of mobile functional near-infrared spectroscopy (fNIRS) monitoring in real-world sports settings to further enhance ecological validity and comprehensively elucidate the brain-cognition-motor coupling mechanisms in authentic competitive contexts.

## Data Availability

The raw data supporting the conclusions of this article will be made available by the authors, without undue reservation.
